# GS-MSDR: Gaussian Splatting with Multi-Scale Deblurring and Resolution Enhancement

**DOI:** 10.3390/s25216598

**Published:** 2025-10-27

**Authors:** Fang Wan, Sheng Ding, Tianyu Li, Guangbo Lei, Li Xu, Tingfeng Ming

**Affiliations:** 1 School of Computer Science, Hubei University of Technology, Wuhan 430068, China; 20021026@hbut.edu.cn (F.W.); 102301188@hbut.edu.cn (S.D.); 20000012@hbut.edu.cn (G.L.); 20040038@hbut.edu.cn (L.X.); 2 College of Engineering, Peking University, Beijing 100091, China; tianyuli@stu.pku.edu.cn; 3 Key Lab of Modern Manufacture Quality Engineering, Hubei University of Technology, Wuhan 430068, China

**Keywords:** imagedegradation, 3D Gaussian Splatting, multi-scale deblurring, resolution enhancement

## Abstract

Recent advances in 3D Gaussian Splatting (3DGS) have achieved remarkable performance in scene reconstruction and novel view synthesis on benchmark datasets. However, real-world images are frequently affected by degradations such as camera shake, object motion, and lens defocus, which not only compromise image quality but also severely hinder the accuracy of 3D reconstruction—particularly in fine details. While existing deblurring approaches have made progress, most are limited to addressing a single type of blur, rendering them inadequate for complex scenarios involving multiple blur sources and resolution degradation. To address these challenges, we propose Gaussian Splatting with Multi-Scale Deblurring and Resolution Enhancement (GS-MSDR), a novel framework that seamlessly integrates multi-scale deblurring and resolution enhancement. At its core, our Multi-scale Adaptive Attention Network (MAAN) fuses multi-scale features to enhance image information, while the Multi-modal Context Adapter (MCA) and adaptive spatial pooling modules further refine feature representation, facilitating the recovery of fine details in degraded regions. Additionally, our Hierarchical Progressive Kernel Optimization (HPKO) method mitigates ambiguity and ensures precise detail reconstruction through layer-wise optimization. Extensive experiments demonstrate that GS-MSDR consistently outperforms state-of-the-art methods under diverse degraded scenarios, achieving superior deblurring, accurate 3D reconstruction, and efficient rendering within the 3DGS framework.

## 1. Introduction

Photo-based 3D reconstruction is a critical field in computer vision, with significant theoretical and practical implications. A key challenge is addressing the deblurring problem in blurry images, which directly affects the accuracy of 3D scene reconstruction. Current research focuses on two main frameworks: neural radiance fields (NeRF) [1] and 3D Gaussian Splatting (3DGS) [2]. NeRF uses implicit representations and differentiable ray tracing to generate high-fidelity view synthesis. However, its volumetric rendering approach is computationally expensive, limiting its efficiency for real-time systems. Although various optimization strategies [3,4,5,6,7,8,9,10,11,12,13] have been proposed to improve the efficiency of neural radiance field (NeRF) frameworks, the inherent computational cost of volumetric rendering remains a major bottleneck. For instance, K-Planes [3] and Plenoxels [4] introduce explicit grid- and plane-based radiance field representations to accelerate convergence by replacing implicit neural networks with lightweight voxel structures. FastNeRF [5] further achieves high-fidelity rendering at real-time frame rates through neural factorization and parallel decomposition, while Instant-NGP [6] employs a multi-resolution hash encoding scheme that significantly enhances spatial indexing efficiency. Direct Voxel Grid Optimization [7] eliminates the need for network-based optimization by directly learning voxel grids, thereby enabling faster and more stable reconstruction. Tensorf [8] leverages low-rank tensor decomposition to reduce the memory footprint and parameter space, whereas Mip-NeRF 360 [9] and Zip-NeRF [10] mitigate aliasing artifacts and improve scalability using anti-aliased, grid-based representations. Additionally, several works extend NeRF to dynamic or generalized scenarios, such as Robust Dynamic Radiance Fields [11], D-NeRF [12], and Non-Rigid NeRF [13], which model non-rigid and temporal variations but introduce additional computational complexity. Despite these advances, volumetric rendering and the high-dimensional optimization inherent to NeRF still demand substantial computational resources, particularly in high-resolution and dynamic scene reconstruction tasks. In contrast, 3DGS employs explicit 3D Gaussian distributions and differentiable rasterization, offering a more efficient solution for high-quality scene reconstruction, particularly for clear images. However, practical applications of 3DGS are often limited by image degradations, such as motion and defocus blur, caused by factors like camera shake and focus shift. These blur effects reduce image clarity and resolution, impairing 3DGS performance in detailed reconstruction and multi-view consistency. The loss of high-frequency information further limits the system’s ability to capture fine details and may cause overfitting during reconstruction, degrading overall quality.

Despite these advantages, standard 3DGS still faces several limitations in practical applications. It assumes sharp and high-quality input images, making it highly sensitive to degradations such as motion and defocus blur. These degradations lead to the loss of high-frequency details and texture consistency across views, causing inaccurate Gaussian parameter estimation and degraded reconstruction fidelity. Moreover, the Gaussian representation inherently smooths fine details during rasterization, which diminishes edge sharpness and texture richness in complex regions. Furthermore, standard 3DGS lacks an adaptive mechanism to capture and enhance degraded features at multiple scales, resulting in reduced robustness and generalization in real-world scenes.

Recent advancements in deblurring for scene reconstruction have been significant. In NeRF, methods like Deblur-NeRF [14] and DP-NeRF [15] improve deblurring through blur kernel estimation and physical priors. PDRF [16] enhances deblurring performance with a two-stage strategy. These methods handle both defocus and motion blur simultaneously. In contrast, other methods [17,18,19,20] focus on a single blur type, such as motion or defocus, and lack adaptability in scenes with multiple concurrent blur types. Additionally, many rely on NeRF’s volumetric framework, making them difficult to apply to 3DGS. In 3DGS, Deblurring 3D Gaussian Splatting [21] introduced the first framework for deblurring using 3D Gaussian point distributions. BAGS [22] uses a blur proposal network (BPN) and a coarse-to-fine strategy to address image blur. Other studies [23,24,25,26,27] primarily focus on optimizing motion blur, with GS-Blur [28] constructing a high-quality dataset specifically for motion blur. While these methods lay the groundwork for 3DGS deblurring, they struggle to handle multiple blur types simultaneously.

Resolution loss is another inherent challenge in 3DGS. This occurs due to the difficulty in capturing high-frequency details during rendering, particularly in complex textures and geometric regions. While Gaussian approximation improves computational efficiency, it inevitably leads to the loss of high-frequency information, weakening 3DGS performance in high-precision reconstruction and reducing the authenticity of visual results. Moreover, standard 3DGS lacks an adaptive mechanism to preserve fine details across varying resolutions, resulting in over-smoothing in dense or edge-rich areas. This limitation becomes more pronounced when input images suffer from motion or defocus blur, as the Gaussian representation cannot effectively recover missing high-frequency components. In real-world scenarios, multiple blur types (e.g., motion, defocus, and downsampling blur) often coexist, further aggravating detail loss and reducing multi-scale resolution. Existing NeRF and 3DGS approaches show limited adaptability in such conditions, compromising the accuracy and realism of reconstructed scenes.

To address the challenges of detail loss and resolution degradation due to image blur in 3DGS, and to overcome the limitations of existing methods in handling multiple blur types, we designed a novel framework—Gaussian Splatting with Multi-scale Deblurring and Resolution Enhancement. The core innovation of this framework is the introduction of a Multi-scale Adaptive Attention Network, which acts as a key feature extraction module, enabling efficient deblurring and resolution enhancement. MAAN improves feature representation in complex scenes by combining shallow feature extraction modules with deep feature fusion mechanisms. The deep feature module utilizes two Feature Attention Networks, which integrate a Global-Aware Kernel Attention and Spatial Adaptive Fusion Module to enhance feature representation across scales. The high-quality RGB-D features extracted by MAAN are then combined with positional encoding and camera embedding information to generate Multi-modal Contextual Features. These multi-modal features capture spatial and perspective information comprehensively, providing a robust foundation for subsequent feature optimization. The MCF is then input into a specially designed Multi-modal Context Adapter for further optimization.

To further enhance reconstruction quality, the GS-MSDR introduces a Hierarchical Progressive Kernel Optimization strategy. HPKO employs a coarse-to-fine optimization strategy: it initially optimizes the blur kernel at low resolution to reduce computational complexity and blur uncertainty, then progressively increases the resolution of both the image and blur kernel to restore finer details at higher resolutions. This progressive optimization mechanism not only addresses detail loss in complex blur scenarios but also significantly reduces computational overhead. By generating adaptive blur kernels and corresponding masks at each layer, HPKO gradually suppresses blurred areas and enhances detail recovery, thereby improving spatial resolution and overall detail performance.

In summary, the contributions of this work are as follows:

- We introduce the GS-MSDR framework, which significantly improves 3D reconstruction results in blurred scenes through multi-scale deblurring and resolution enhancement.
- We propose the Multi-scale Adaptive Attention Network (MAAN), which enables the extraction of multi-scale features and selective feature enhancement.
- We demonstrate the effectiveness of GS-MSDR through experiments in three types of image blur scenarios, showing significant improvements in both quantitative metrics and visual effects compared to current state-of-the-art methods.

## 2. Related Works

### 2.1. Image Deblurring

Image deblurring is a fundamental task in image restoration, addressing blurring effects caused by factors such as camera shake, object motion, and lens defocus [29,30]. These blur effects degrade image quality and severely impact the ability to resolve scene details, especially in computer vision and graphics applications requiring high-precision reconstruction.

Image blur typically manifests in two types: motion blur, resulting from camera or object movement, and defocus blur, caused by out-of-focus objects. These types exhibit distinct characteristics and impact the image content differently. To model these blur types, a generative process using joint blur kernels and pixel distribution weights can be formulated as Equation (1):

(1) $$G\left(x\right)=\int_{S\in R^{2}}K\left(x,s\right)\cdot W\left(x-s\right)f\left(x-s\right)\;ds+\eta \left(x\right),$$

where G(x) represents the blurred image, K(x) is the blur kernel, W(x) is the pixel distribution weight, and η(x) models noise in real-world imaging processes.

Traditional deblurring methods rely on inverse optimization, using deconvolution techniques such as Wiener filtering and Richardson–Lucy deconvolution [31,32,33,34,35]. However, these methods struggle with inaccuracies in noise and blur kernel estimation. Recent deep learning approaches, particularly convolutional neural networks (CNNs), have proven effective for end-to-end training in addressing complex blurs. Methods like Deblur-GAN [36,37] and Deep-Deblur [38,39] have shown notable success. Despite these advancements, previous methods are primarily designed for two-dimensional scenarios and do not fully account for the consistency required in 3D scenes, limiting their applicability in 3D scene deblurring tasks.

### 2.2. Neural Radiance Fields (NeRF)

NeRF [1] is a novel 3D reconstruction method that generates high-quality 3D scenes from 2D images by implicitly encoding the scene’s volumetric information using deep neural networks. It employs a multi-layer perceptron (MLP) [1] to map points in 3D space to radiance field information from different viewpoints, enabling high-precision representations of color and density. By learning implicit functions of spatial coordinates and viewpoint directions, NeRF generates realistic rendered images from multiple viewpoints, greatly advancing scene reconstruction and viewpoint synthesis in computer vision and graphics. This method relies on volumetric rendering, sampling along light rays, and accumulating density and color information at each point to generate high-quality images. It excels in handling complex scene details and lighting effects and is widely applied in virtual reality, augmented reality, robotic navigation, among other domains. However, its reliance on dense sampling significantly increases computational costs, limiting its applicability in real-time rendering.

To enhance rendering efficiency, fast neural rendering has employed various acceleration strategies. In recent years, researchers have substantially reduced the computational complexity of MLP and improved inference speed by introducing data structures such as mesh structures [3,4,7,8,40,41,42,43] and hash tables [6,10]. Additionally, techniques such as pre-training and parameter compression have been applied to accelerate rendering and meet stricter real-time requirements. However, rendering efficiency continues to face challenges in high-resolution and complex scenes. The latest 3D-GS [2] method leverages explicit 3D Gaussian distributions and differentiable rasterization techniques to further enhance rendering speed, enabling high-quality image generation in real-time scenarios.

### 2.3. NeRF-Based Deblurring

NeRF-based deblurring methods integrate deblurring techniques with 3D scene reconstruction to extract clear 3D structures from blurred inputs. Approaches such as Deblur-NeRF [14] and DP-NeRF [15] use blur kernel estimation and physical priors to recover motion-blurred scenes. BAD-NeRF [17] incorporates camera motion trajectory learning, making reconstruction results more consistent with real-world blur processes. However, most of these methods depend on implicit MLP structures and volumetric rendering, limiting their real-time deblurring efficiency. Furthermore, these methods typically address only one blur type, such as motion blur or defocus blur.

Other techniques, such as Sparse-DeRF [44], mitigate overfitting under sparse view conditions using geometric constraints and perceptual distillation, improving structural and perceptual quality. However, its complex optimization process limits its applicability. Deblur e-NeRF [19] handles motion blur in high-speed and low-light conditions using event camera models, but it incurs high computational costs. LSE-NeRF [20] improves deblurring performance by using temporal embedding and gamma mapping to correct RGB and event camera response differences. However, its reliance on dual-modal data limits practicality in general scenarios.

In contrast, the proposed deblurring framework based on 3D Gaussian Splatting leverages explicit 3D Gaussian distributions and rasterization to achieve efficient deblurring in real-time, generating high-quality images in complex scenes and adapting to various types of blur.

### 2.4. 3DGS-Based Deblurring

In the domain of 3D Gaussian Splatting, recent research has explored explicit Gaussian-based representations for efficient and high-quality deblurring. Deblurring 3D Gaussian Splatting introduced the first framework for deblurring using 3D Gaussian point distributions. BAGS uses a blur proposal network and a coarse-to-fine strategy to address image blur. Other studies primarily focus on optimizing motion blur, with GS-Blur constructing a high-quality dataset specifically for motion blur. Despite these advances, most 3DGS-based deblurring methods remain limited to single-type degradations (e.g., motion or defocus blur) and often lack adaptability to mixed or scale-dependent blur conditions. Moreover, traditional Gaussian Splatting lacks adaptive mechanisms for multi-scale feature extraction and high-frequency detail recovery, resulting in reduced performance in real-world complex scenes.

By combining multi-scale feature learning with explicit Gaussian primitives, GS-MSDR bridges the gap between 3DGS-based reconstruction and multi-scale deblurring. This design allows for the simultaneous enhancement of visual fidelity and computational efficiency, achieving superior performance in both quantitative metrics and perceptual quality compared to existing 3DGS-based approaches.

## 3. Methodology

### 3.1. Preliminaries: Three-Dimensional Gaussian Splatting

Three-dimensional Gaussian Splatting (3D-GS) [2] is a technique for representing 3D scenes using explicit point clouds and rendering them via differentiable rasterization techniques. This approach enables efficient 3D reconstruction and novel view synthesis. In this method, the scene is modeled as a set of 3D Gaussian distributions, where each Gaussian represents a small volumetric element of the scene. Specifically, each 3D Gaussian distribution is defined by its position $x\in R^{3}$, covariance matrix $\Sigma \in R^{3\times 3}$, color $c\in R^{3}$, and opacity α∈R. The probability density function of a Gaussian distribution at position *x* is expressed as shown in Equation (2):

(2) $$G\left(x\right)=e^{-\frac{1}{2}{\left(x-\mu \right)}^{\;T}\Sigma^{-1}\left(x-\mu \right)},$$

where (μ) represents the mean of the Gaussian distribution (i.e., the 3D position), and Σ is the covariance matrix that controls the shape, direction, and scale of the Gaussian.

To ensure the covariance matrix is physically valid, 3D-GS decomposes Σ into a rotation matrix $R\in R^{3\times 3}$ and a scaling matrix $S\in R^{3\times 3}$, as given in Equation (3):

(3) $$\Sigma =RSS^{T}R^{T},$$

where R represents the rotation of the 3D Gaussian in space, and S controls the scaling variations along the principal axes.

During rendering, 3D Gaussian Splatting generates images by projecting each 3D Gaussian onto a 2D image plane. Given a camera pose $T=\{R_{c},t_{c}\}$, 3D-GS transforms the covariance matrix Σ of each 3D Gaussian into the covariance matrix $\Sigma^{\prime }$ on the 2D image plane, as detailed by Equation (4).

(4) $$\Sigma^{\prime }=JWZW^{T}J^{T},$$

where W is the view transformation matrix, J is the Jacobian matrix approximating the projection transformation, and Z accounts for the scaling factor.

To calculate the color of each pixel, 3D-GS employs a depth-sorted alpha blending method. Specifically, the overlapping regions of *N* depth-sorted 2D Gaussians at each pixel are combined using Equation (5).

(5) $$C=\sum_{i=1}^{N}c_{i}\alpha_{i}\prod_{j=1}^{i-1}\left(1-\alpha_{j}\right).$$

where $c_{i}$ represents the color of the *i*-th Gaussian distribution, and $\alpha_{i}$ denotes the alpha value derived from the opacity of the projected 2D Gaussian and its density. Additional details can be found in [2].

### 3.2. Multi-Scale Adaptive Attention Network

The Multi-scale Adaptive Attention Network (MAAN) serves as the principal feature extraction and resolution enhancement module in the proposed GS-MSDR framework. It is designed to jointly perform multi-scale deblurring and resolution enhancement under the 3D Gaussian Splatting (3DGS) framework through an end-to-end optimization process. Rather than operating sequentially, the MAAN module is tightly integrated into the Gaussian Splatting pipeline, where gradients are jointly propagated across both deblurring and resolution enhancement components to ensure consistent optimization and mutual reinforcement between them. This integration allows MAAN to maintain high efficiency and stability during the training and rendering process.

**Figure 1:**
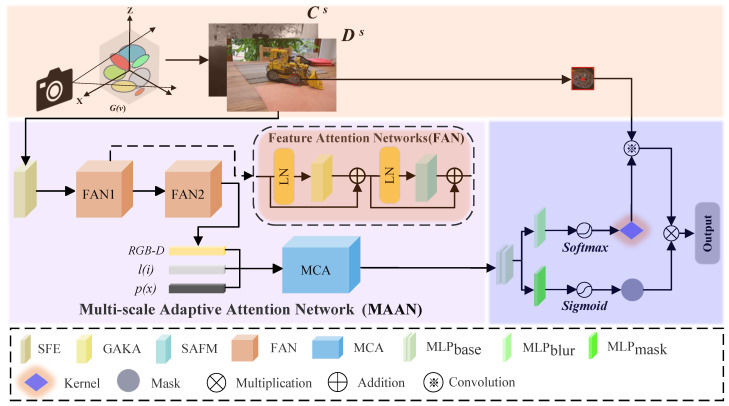
Overall architecture of the proposed GS-MSDR framework. The system jointly performs multi-scale deblurring and resolution enhancement within the 3D Gaussian Splatting (3DGS) pipeline. RGB-D features, along with positional and view embeddings, are processed through the Feature Attention Network (FAN) and Multi-scale Adaptive Attention Network (MAAN) for feature refinement. The final layer applies sigmoid and softmax functions to estimate blur kernels and masks, producing high-fidelity scene reconstructions.

MAAN incorporates an adaptive attention mechanism that captures both global contextual information and fine-grained spatial details, thereby improving feature representation across multiple scales. Structurally, MAAN consists of three key components: the Feature Attention Network (FAN), the Multi-modal Contextual Features (MCF), and the Multi-modal Context Adapter (MCA). The FAN adaptively refines spatial features using hierarchical attention, while the MCF fuses multi-modal inputs such as RGB-D, positional, and camera embedding information to form a unified representation. The MCA further optimizes these features through context-aware refinement, enabling robust reconstruction even under complex blur and resolution degradation conditions. The overall pipeline is illustrated in Figure 1.

#### 3.2.1. Feature Attention Network

**Figure 2:**
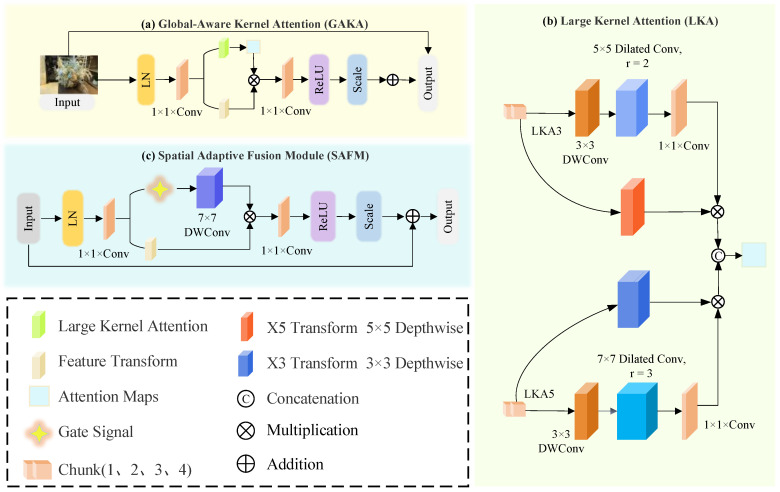
Structure of the Feature Attention Network (FAN). The FAN integrates three sub-modules: (**a**) Global-Aware Kernel Attention (GAKA) for multi-scale contextual extraction, (**b**) Large Kernel Attention (LKA) for capturing long-range dependencies, and (**c**) Spatial Adaptive Fusion Module (SAFM) for selective spatial feature enhancement. These modules collaboratively improve feature representation and deblurring capability.

The Shallow Feature Extractor (SFE) utilizes a 3×3 convolution to generate a low-level feature map that captures essential edge and texture information. The Feature Attention Network (FAN) employs both global and local attention mechanisms to enhance feature representation across scales [45,46,47,48,49]. It integrates the proposed Global-Aware Kernel Attention (GAKA) and Spatial Adaptive Fusion Module (SAFM), as shown in Figure 2. GAKA captures multi-scale contextual information via convolutional kernel decomposition, while SAFM focuses on critical spatial regions, balancing global context and local detail.

In image deblurring tasks, capturing both detailed and broad features is crucial. The proposed GAKA serves as the cornerstone of the FAN. GAKA captures global, multi-scale information via feature extraction and adaptive recalibration, while reducing computational complexity through convolutional kernel decomposition. This ensures both efficiency and enhanced representational capability.

To stabilize feature distribution, standardization is first applied, followed by a 1×1 convolution that maps the *n*-dimensional feature space to 2n-dimensional. The projected features are split into Large Kernel Attention (LKA) and Feature Transform branches, balancing local and global feature extraction. LKA captures long-range dependencies using decomposed convolutions. Two substructures, LKA3 and LKA5, are alternately applied to process spatial dependencies at varying scales. The LKA computation is defined as Equation (6):

(6) $$\mathrm{LKA}\left(X\right)=f_{PW}\left(f_{DWD}\left(f_{DW}\left(X\right)\right)\right).$$

where $f_{DW}$ denotes depth-wise convolution, which is used for preliminary global feature extraction, and $f_{DWD}$ represents dilated convolution, which refines global information. Finally, $f_{PW}$ denotes point-wise convolution, used for feature fusion. This decomposition approach ensures that global dependencies are captured while reducing computational complexity, a key advantage of the GAKA module in handling complex scenarios.

To enhance discriminative power, parallel transformation paths X3 and X5 apply 3×3 and 5×5 depth-wise separable convolutions with attention-based recalibration. The resulting multi-scale features are fused and modulated through a 1×1 convolution, ReLU, and learnable scaling. SAFM further refines global features, focusing on critical regions. Input features are normalized via LayerNorm, expanded to 2n channels, and split into gating and feature branches. The gating path uses a 7×7 depth-wise separable convolution for spatial context extraction. Adaptive modulation is performed through element-wise multiplication, followed by dimensionality reduction, ReLU, and residual scaling, producing the enhanced output. This design balances global context modeling and computational efficiency, enabling robust performance in complex image deblurring scenarios.

#### 3.2.2. Multi-Modal Contextual Features

To distinguish between scenes and blurs, the MCF is designed for addressing the variability of real image blurs across different images and pixels. A learnable view embedding l(i) and position embedding p(x) are introduced for training the view *i* and coordinate x. RGB-D features extracted from rendered images and depth maps through FAN are combined with these embeddings to form the MCF in MAAN. This integration allows MAAN to effectively capture local statistical information, particularly in areas with strong blur, such as edges and corners. The MCF can be formulated as shown in Equations (7) and (8):

(7) $$\begin{array}{cc} f_{\mathrm{RGB}\text{-}D}\left(x,i\right) & =G_{\mathrm{FAN}}\left(C(x,i)⊕D(x,i)\right), \end{array}$$

(8) $$\begin{array}{cc} k(x,i),\;m(x,i) & =G_{\mathrm{kernel}}\left(l\left(i\right)⊕p\left(x\right)⊕f_{\mathrm{RGB}\text{-}D}\left(x,i\right)\right). \end{array}$$

where C(x,i) and D(x,i) denote color and depth, and ⊕ denotes concatenation.

Additionally, scene depth is closely linked to blur degree. In defocus blur, blur increases with distance from the focal plane, while in camera motion blur, foreground pixel displacement is more pronounced than background displacement. Incorporating RGB-D features enhances MAAN’s modeling capability, improving new view synthesis quality.

#### 3.2.3. Multi-Modal Context Adapter

**Figure 3:**
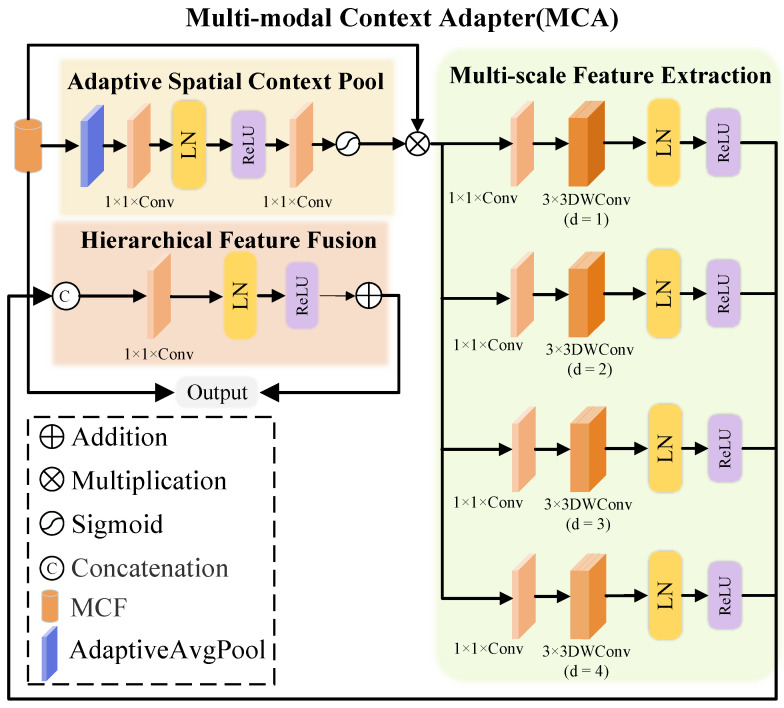
Structure of the Multi-modal Context Adapter (MCA). The MCA extracts hierarchical multi-scale features using depth-wise convolutions and fuses them through adaptive spatial context pooling. Element-wise operations and sigmoid activation integrate RGB-D, positional, and camera embeddings, enhancing context understanding and reconstruction accuracy.

MCA enhances the model’s ability to restore details and structures in blurry scenes using a hierarchical multi-scale adaptive mechanism. MCA handles complex blur and multi-scale information by employing adaptive spatial context pooling, multi-scale feature extraction, and hierarchical feature fusion, progressively optimizing feature representation from global to local, improving new view synthesis quality, as illustrated in Figure 3. Adaptive spatial context pooling captures global context and assigns spatial region-specific weights. It aggregates global features from the MCF using adaptive average pooling, followed by 1×1 convolution layers, layer normalization, ReLU activation, and Sigmoid activation to generate dynamic spatial weights.

MCA further captures details at various scales by splitting input features into four parallel branches, each applying convolutions with increasing dilation rates to extract features at different scales. The processing of the *i*-th branch is given by Equations (9) and (10):

(9) $$B_{i}=\mathrm{ReLU}\left(\mathrm{LN}\left({\mathrm{Conv}}_{3\times 3,\mathrm{dilation}=i}\left(X_{i}\right)\right)\right),\;i=1,\ldots ,4,$$

where the dilation rate *i* ranges from 1 to 4 and captures fine details at a global context. This ensures sensitivity to both local details and global structure across scales, facilitating robust multi-scale information fusion.

Finally, the original input MCF is added to the fused feature $F_{fused}$ via a residual connection to yield the final output:

(10) $$F_{\mathrm{MCA}}=F_{\mathrm{fused}}+X.$$

This residual connection preserves original input information while enhancing the expressiveness of the fused features, significantly improving detail clarity, edge sharpness, and structural recovery.

### 3.3. Hierarchical Progressive Kernel Optimization

In 3D scene reconstruction tasks, high-quality deblurring relies on accurate blur kernel estimation. However, directly optimizing the blur kernel at high resolution incurs high computational costs and may lead to unstable training [14,15]. To address this, we propose HPKO, a method that enhances image and blur kernel resolution progressively, enabling coarse-to-fine optimization. This approach improves blur kernel accuracy while maintaining computational efficiency, offering an effective solution for blur processing in 3D reconstruction. The HPKO settings for the optimization process are summarized in Table 1.

HPKO’s core is its hierarchical strategy, which refines image and blur kernel resolution layer by layer. Initial blur kernel estimation at a lower resolution reduces computational costs and uncertainty. As the hierarchy progresses, HPKO refines the blur kernel at each stage, enhancing image clarity and detail. Blur kernels and masks are estimated at each hierarchical scale *s* (with downsampling factor $2^{s-1}$), allowing for adaptive modeling across resolutions. At each scale, intermediate features $f_{\mathrm{mid}}^{s}\left(x,i\right)$ are computed by integrating view, positional, and RGB-D embeddings, as described in Equations (11), (12) and (13).

(11) $$\begin{array}{cc} f_{\mathrm{mid}}^{s}\left(x,i\right) & =M_{\mathrm{base}}^{s}\left(l\left(i\right)⊕p\left(x\right)⊕f_{\mathrm{RGB}\text{-}D}\left(x,i\right)\right), \end{array}$$

(12) $$\begin{array}{cc} k^{s}\left(x,i\right) & =\mathrm{softmax}\left(M_{\mathrm{blur}}^{s}\left(f_{\mathrm{mid}}^{s}\left(x,i\right)\right)\right), \end{array}$$

(13) $$\begin{array}{cc} m^{s}\left(x,i\right) & =\mathrm{sigmoid}\left(M_{\mathrm{mask}}^{s}\left(f_{\mathrm{mid}}^{s}\left(x,i\right)\right)\right). \end{array}$$

Here, $M_{\mathrm{blur}}^{s}$ and $M_{\mathrm{mask}}^{s}$ are scale-specific multi-layer perceptrons that output blur kernels and masks, respectively. This hierarchical structure enables the efficient handling of various blur types and resolutions, promoting effective detail recovery in high-fidelity scene reconstruction.

Compared to traditional methods, HPKO’s progressive optimization reduces computational complexity while significantly improving the model’s ability to capture details in blurred regions. It ensures consistent performance across scales, especially in high-resolution reconstruction tasks, and prevents over-smoothing in severely blurred areas. Overall, HPKO improves 3D scene clarity while maintaining computational efficiency, offering an effective solution for complex scene deblurring.

## 4. Experiments

### 4.1. Implementation Details

The proposed method was implemented using PyTorch 1.12.1 within the 3D-GS framework, and we present the training, testing time, and GPU memory usage of the proposed method for three different scenarios, with the results summarized in Table 2. To optimize the Gaussian function, we utilized the Adam optimizer [50] with a learning rate identical to that used in the original 3D-GS model. Multi-scale training was conducted with three scales, starting at s=3, and parameters set as $K_{3}=5$, $K_{2}=9$, and $K_{1}=17$. GS-MSDR demonstrated strong performance across multiple resolutions. Camera poses and Gaussian distributions, obtained using COLMAP [51], were used for initialization. All experiments were executed on an NVIDIA RTX 4090 GPU (NVIDIA Corporation, Santa Clara, CA, USA).

In addition to runtime and memory analysis, we also evaluated the scalability of the proposed method across different input resolutions and scene complexities. The method demonstrates stable performance and linear scalability with respect to both image resolution and dataset size. As the input resolution increases from 1× to 4×, the computational cost grows proportionally, while the reconstruction quality remains consistent. This indicates that the proposed hierarchical optimization and adaptive kernel design effectively maintain efficiency under larger-scale or more complex scenarios, highlighting the method’s robustness and applicability to real-world 3D reconstruction tasks.

### 4.2. Benchmark Datasets

For evaluating camera motion blur and defocus blur, we used the Deblur-NeRF dataset (ETH Zurich, Zurich, Switzerland) [14], which includes real-world scenes spanning ten different scenarios for each blur type. Experiments were conducted to assess the impact of inconsistent input image resolutions using the Mip-NeRF 360 dataset (Google Research, Mountain View, CA, USA) [9], which contains nine distinct scenes. Specifically, the training images were divided into four equal parts, each corresponding to different downscaling factors of 4×, 3×, 2×, and no downscaling (1×). The image splits were sampled randomly and uniformly to ensure that no downscaled segment spanned consecutive intervals of the camera trajectory. This random sampling strategy ensured that the downsampling process was unbiased with respect to specific camera positions, thereby enhancing the robustness of the experiment.

Following the downsampling, we used NeRFStudio’s data processing pipeline [52] to convert all images to the highest resolution and calibrate them using COLMAP [51]. In line with previous works [9,10,53], the entire process was conducted on downscaled data rather than directly using the original 4K resolution images. This approach was chosen to simulate real-world conditions where images may have varying resolutions, ensuring the experiment reflects realistic scenarios and avoids overfitting to high-quality, uniformly resolved data.

In terms of the downscaling and upscaling processes, the training images were first downsampled to different resolutions (4×, 3×, 2×, and 1×). For example, the 4× downscaling was achieved by reducing the original image by a factor of four, with similar operations applied for the other resolutions. After downsampling, we then applied NeRFStudio’s pipeline to upscale all images to their highest resolution, which is the original 4K resolution. This upsampling ensures that all images used in training have the same resolution, maintaining consistency throughout the experiment. Therefore, the evaluation phase was based on processed images, which were downsampled and then upscaled, rather than directly using the original 4K resolution images.

For the training process, we adopted a multi-scale optimization scheme. The optimization started with lower resolution images and gradually increased the resolution. Initially, we used a smaller kernel size, and as the resolution increased, the kernel size was adjusted. This gradual increase in resolution helped stabilize the optimization process, preventing overfitting to sparse data. The adjustment of kernel sizes ensured that each resolution group’s kernel size was consistent with the level of blur in the images, ultimately improving the final reconstruction quality.

### 4.3. Baselines and Metrics

The proposed method was compared with several state-of-the-art deblurring techniques in neural rendering and 3D Gaussian models: 3D-GS [2], Deblur-NeRF [14], DP-NeRF [15], PDRF [16], Sharp-NeRF [18], BAGS [22] and Mip-Splatting [53]. Performance was evaluated on the benchmark Deblur-NeRF dataset [14], which includes both synthetic and real images with camera motion blur or defocus blur. For mixed resolutions, we also evaluated Mip-NeRF 360 [9], NeRFacto [52], Mip-Sp [53], and Sp-HAT [54].

The proposed method was assessed using the Mip-NeRF 360 dataset [9]. To quantitatively assess reconstruction quality, we employed two commonly used image fidelity metrics—Peak Signal-to-Noise Ratio (PSNR) and Structural Similarity Index Measure (SSIM). PSNR evaluates the pixel-wise reconstruction accuracy by measuring the logarithmic ratio between the maximum possible signal power and the power of the noise introduced by reconstruction errors. A higher PSNR value indicates that the reconstructed image is closer to the ground truth, reflecting higher overall fidelity. SSIM, on the other hand, measures the perceived structural similarity between the reconstructed and reference images by considering luminance, contrast, and structural information. Unlike PSNR, which focuses solely on pixel-level differences, SSIM aligns better with human visual perception, making it particularly effective in assessing texture preservation and structural consistency in 3D reconstruction tasks.

These two metrics were selected because they are widely adopted in both NeRF-based and 3D Gaussian Splatting-based rendering studies, ensuring fair and consistent comparisons with existing methods. Furthermore, PSNR and SSIM together provide a balanced evaluation of both numerical accuracy and perceptual quality, which are essential for assessing deblurring and resolution enhancement performance in high-fidelity 3D scene reconstruction.

### 4.4. Computational Complexity and Trade-Off Analysis

Compared with the standard 3D Gaussian Splatting (3DGS), the proposed GS-MSDR introduces additional modules, including the Multi-scale Adaptive Attention Network (MAAN) and the Hierarchical Progressive Kernel Optimization (HPKO), to enhance deblurring and resolution restoration. Although these components slightly increase the overall computational complexity, the additional cost is moderate and well justified by the performance improvement.

As summarized in Table 2, the training time of GS-MSDR ranges from approximately 1.0 to 2.0 h depending on the blur type, while testing requires only 6–12 s per scene. The GPU memory utilization on an NVIDIA RTX 4090 varies between 45% and 75%, which demonstrates that the proposed method remains computationally efficient and scalable.

The marginal increase in computational cost mainly arises from the adaptive attention and kernel optimization operations, which contribute significantly to fine-detail recovery and multi-scale feature refinement. Despite this increase, GS-MSDR achieves a favorable balance between efficiency and accuracy, yielding substantial gains in PSNR and SSIM metrics (Table 3, Table 4 and Table 5). Therefore, the proposed framework maintains practical efficiency while providing superior reconstruction quality and robustness in complex blurred scenarios.

## 5. Results

### 5.1. Ablation Study

To evaluate the contribution of each module in our proposed model, we conducted a series of ablation experiments to assess the impact of removing individual components on the overall model performance. The experiments focused on three common types of image blur: camera motion blur, true defocus blur, and hybrid resolution blur. We employed PSNR and SSIM as the primary performance metrics to quantify image restoration quality. The experimental results are summarized in Table 6.

In the absence of the GAKA module, the model’s performance deteriorates significantly across all blur types. As depicted in Table 6, particularly under true defocus and camera motion blur conditions, the recovery of image details is severely impaired, and the restoration is insufficient. These findings underscore the critical role of the GAKA module in enabling efficient image feature extraction and restoration. Without it, the model is unable to effectively recover fine details, especially in challenging blur scenarios.

Removing the SAFM module results in a notable decline in model performance when handling true defocus blur. Both PSNR and SSIM values experience significant reductions, as shown in Table 6. The absence of this module severely limits the model’s recovery capability under complex blur conditions, highlighting the essential role of the SAFM module in image reconstruction. It is particularly instrumental in optimizing the restoration of images with intricate blur characteristics, as it helps preserve fine details and texture in defocused areas.

The removal of the MCA module leads to decreased performance, particularly under mixed resolution blur conditions. Table 6 illustrates that, in the absence of this module, the model’s ability to adapt to varying resolutions and blur levels is compromised, resulting in suboptimal recovery. The MCA module plays a pivotal role in enhancing the model’s robustness through the fusion of multi-scale features, thereby improving its adaptability and performance across different blur types and resolution variations.

When the RBGD module is excluded, a slight degradation in performance is observed, especially in scenarios involving true defocus and mixed resolution blur. As shown in Table 6, the RBGD module contributes to improved image restoration by enhancing the accuracy of blur kernel modeling. This is particularly evident in complex blur situations where precise blur modeling is essential for effective recovery.

Finally, the fully integrated HPKO model, which combines all the modules, achieves the best performance across all blur types. Table 6 demonstrates that the PSNR and SSIM values are highest under camera motion blur, significantly outperforming the results of other methods. This highlights the HPKO model’s ability to restore high-quality images across a wide range of blur scenarios, owing to its optimization of both image features and blur kernel parameters.

Overall, the ablation experiments clearly illustrate that each module contributes significantly to the overall performance of the model. Among these, the HPKO module, which integrates multiple optimization strategies, plays a central role in enhancing the model’s ability to handle complex blur conditions. The introduction of each module progressively improves the model’s restoration capability, culminating in a robust framework capable of effectively dealing with various types of blur and restoring high-quality images.

#### 5.1.1. Functional Component Analysis

To further quantify the contribution of the two key functional components—multi-scale deblurring (HPKO) and resolution enhancement (MAAN)—we conducted an additional ablation experiment. This experiment isolates each mechanism and evaluates their individual and combined impact on performance, as summarized in Table 7.

The results indicate that both HPKO and MAAN independently contribute to image restoration, but their combination yields the highest improvement. HPKO primarily enhances deblurring performance by progressively optimizing blur kernels across scales, while MAAN effectively restores fine texture and edge details through adaptive attention-based feature refinement. The integrated GS-MSDR framework combines these benefits, achieving a PSNR gain of up to +2.18 dB and an SSIM improvement of +0.09 over the baseline, confirming the complementary and synergistic nature of the two modules.

#### 5.1.2. Cross-Module Interaction Analysis

To further explore module sensitivity and interdependency, we analyzed the performance impact of selectively removing one module at a time while keeping HPKO active. The results, shown in Table 8, reveal the degree of inter-module cooperation and the stability of the system under partial module removal.

The findings demonstrate strong cross-module synergy within the GS-MSDR architecture. Removing any sub-module results in a measurable performance drop, but the degree of decline varies by function. The RGBD module exhibits the highest sensitivity, confirming the importance of joint RGB–depth feature modeling in handling diverse degradations. SAFM removal causes the largest performance loss in motion blur, underscoring its spatial gating and attention fusion capability. MCA contributes primarily to structural coherence under mixed resolution conditions, while GAKA ensures global contextual consistency. Overall, the results validate that the GS-MSDR’s effectiveness arises from collaborative optimization between modules rather than independent feature processing.

In addition to the standard ablation analysis presented in Table 6, the extended experiments (Table 7 and Table 8) reinforce the technical soundness of the GS-MSDR design. The synergy between MAAN and HPKO proves crucial for stable optimization and high-fidelity scene reconstruction, highlighting the model’s robust adaptability to multi-type image degradations.

### 5.2. Contrast Experiment

In this section, we present both qualitative and quantitative experimental results, providing a comprehensive evaluation of the proposed method. The evaluation framework employs multiple metrics to assess the effectiveness of our approach. Initially, established metrics such as PSNR and SSIM were used.

As shown in Table 3, the proposed method significantly outperforms other models in terms of PSNR and SSIM for the camera motion blur scenario, achieving state-of-the-art performance. Table 4 indicates that the proposed method achieves PSNR comparable to state-of-the-art models in defocus blur scenarios across all metrics. Several factors explain the less noticeable improvement: First, exposure differences between the collected ground truth and training images reduce the reliability of image similarity metrics such as PSNR. Second, many scenes contain specular reflections, leading to suboptimal reconstruction for all methods. Nevertheless, the proposed method achieves state-of-the-art performance under SSIM. As shown in Table 5, in mixed resolution scenarios, the proposed method outperforms all other methods, achieving an average PSNR of 28.30 and an SSIM of 0.819, both exceeding previous methods.

**Figure 4:**
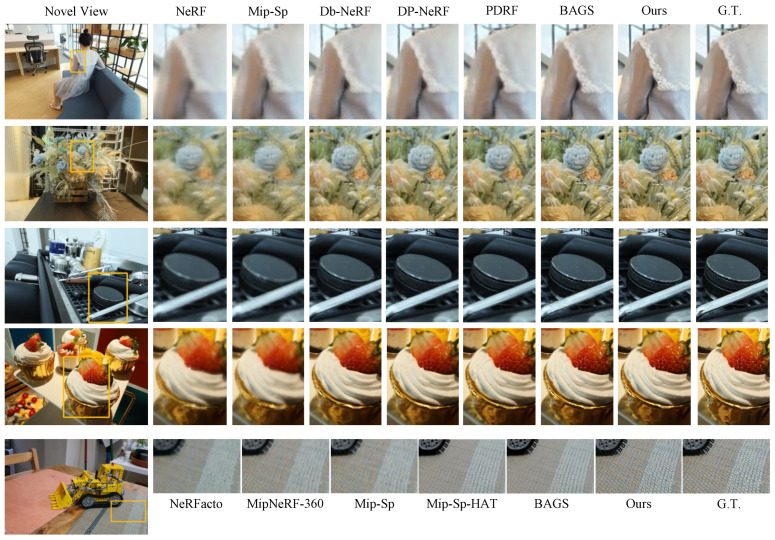
Visualizations of test views on camera motion, defocus blur, and mixed resolution datasets. Mip-Sp and Db-NeRF are short for Mip-Splatting [53] and Deblur-NeRF [14].

We present the qualitative results in Figure 4. Compared to previous methods, the proposed approach yields clearer results when handling the three types of blur discussed above. While overall visual quality improves significantly, the proposed method excels in detail restoration, demonstrating outstanding advantages. This precise detail recovery renders the results nearly indistinguishable from the ground truth images. These findings indicate that the proposed method has a significant advantage in handling complex, blurred scenes.

## 6. Discussion

Image blur can be interpreted as a spatial low-pass filtering process that suppresses high-frequency components responsible for edges, fine textures, and micro-geometry. The proposed GS-MSDR counteracts this effect by explicitly decomposing feature learning across scales. The Multi-scale Adaptive Attention Network (MAAN) exposes multiple receptive fields to the network through attention-based modules, while the Hierarchical Progressive Kernel Optimization (HPKO) progressively refines blur kernels from coarse to fine. This hierarchical strategy stabilizes optimization at low resolutions and gradually restores high-frequency structures at higher scales. As a result, the model effectively recovers fine details and texture sharpness while maintaining robustness in complex degraded scenarios.

The effectiveness of multi-scale processing can also be interpreted from the perspective of human visual perception. The human visual system (HVS) processes information through multi-scale spatial-frequency channels, exhibiting different sensitivities across frequency bands. Perceptual sharpness depends primarily on mid-to-high frequency contrast, while global scene organization relies on low-frequency content. MAAN’s scale-selective attention mimics this perceptual mechanism by allocating higher weights to features that enhance local contrast and edge integrity, while suppressing redundant or blur-consistent responses. Similarly, the adaptive spatial context pooling in the Multi-modal Context Adapter (MCA) performs a scene-dependent gain control that amplifies visually informative regions, such as edges and textured areas. This explains the notable improvement in perceptual quality, as reflected by the superior SSIM values and consistent PSNR gains across all test scenarios.

Empirically, GS-MSDR demonstrates significant improvements in both quantitative and qualitative performance. It consistently achieves higher PSNR and SSIM scores across motion blur, defocus blur, and mixed resolution datasets, indicating enhanced pixel-level fidelity and perceptual realism. The visual comparisons further confirm that GS-MSDR restores sharper boundaries, clearer textures, and improved depth consistency, achieving reconstruction quality closer to human visual perception. These results validate the effectiveness of integrating multi-scale deblurring and resolution enhancement within the 3D Gaussian Splatting framework.

Although the introduction of multi-scale processing and attention mechanisms slightly increases computational cost, as discussed in Section 4.4, this trade-off is moderate and well justified by the substantial gains in reconstruction quality. The framework maintains practical efficiency, with training and testing times remaining within acceptable limits. Nevertheless, under extreme degradations such as severe motion or heavy defocus, high-frequency information may be largely missing, leading to local over-sharpening or reconstruction ambiguity. Addressing such cases will require incorporating stronger physical priors and uncertainty-aware mechanisms in future work.

Overall, the proposed GS-MSDR framework successfully bridges computational modeling and perceptual principles. By aligning multi-scale feature extraction with human visual characteristics, it achieves a favorable balance between reconstruction accuracy, perceptual fidelity, and computational efficiency, making it a robust and scalable solution for real-world 3D reconstruction under complex degradation conditions.

## 7. Conclusions

We proposed GS-MSDR, a novel method to address multi-type image blur and resolution degradation in 3D scene reconstruction. Existing neural radiance field (NeRF)-based approaches often fail to cope with blur or low-resolution images, limiting their utility in complex, real-world scenarios. GS-MSDR incorporates a Multi-scale Adaptive Attention Network (MAAN) that significantly enhances reconstruction quality by capturing fine-grained scene details and adapting to diverse image degradations, such as motion blur, defocus blur, and mixed resolutions. Our framework employs a comprehensive multi-scale feature processing mechanism and a Hierarchical Progressive Kernel Optimization (HPKO) strategy, which together improve both reconstruction accuracy and computational efficiency through an end-to-end optimization process.

Extensive experiments on multiple benchmark datasets demonstrate that GS-MSDR outperforms state-of-the-art methods in both quantitative metrics (PSNR and SSIM) and qualitative visual fidelity across various degradation scenarios. The proposed framework not only advances the deblurring capability within the 3D Gaussian Splatting paradigm but also provides a unified solution that effectively balances reconstruction quality and computational cost. These results validate the effectiveness of integrating multi-scale feature learning and hierarchical optimization for robust 3D reconstruction under complex real-world degradations.

Beyond the benchmark datasets, GS-MSDR has broad potential applications in practical domains such as 3D cultural heritage reconstruction, robotics, and virtual or augmented reality. For instance, it can be applied to the digital preservation of cultural artifacts captured under suboptimal imaging conditions, or to improve visual mapping in autonomous systems affected by motion-induced blur. Moreover, its ability to enhance multi-scale detail and spatial consistency makes it promising for immersive rendering and real-time visualization in virtual environments.

Nevertheless, several limitations remain. GS-MSDR does not explicitly address other real-world degradations such as lighting inconsistencies, occlusions, or rolling-shutter distortions, which may occur in uncontrolled capture settings. In extremely degraded scenes where high-frequency details are largely absent, the network may still produce locally over-sharpened regions or lose fine texture information. Additionally, the method assumes accurate camera pose estimation, and significant pose errors may affect cross-view consistency.

In future work, we plan to extend GS-MSDR to handle more challenging degradation factors, such as illumination variation and dynamic occlusions, and to integrate rolling-shutter compensation for handheld camera data. Further efforts will focus on enhancing feature extraction efficiency to support real-time rendering and adapting the framework to large-scale scene reconstruction in multimedia and robotics applications. Overall, GS-MSDR provides an effective and scalable foundation for high-fidelity 3D reconstruction, paving the way for future developments in perceptually aligned and physically grounded neural rendering systems.

## Figures and Tables

**Table 1:** HPKO settings for the optimization process.

Parameter	Value/Description
Number of Scales	3 (Small, Medium, Large)
Kernel Sizes	$K_{1}=5$ (Small), $K_{2}=9$ (Medium), $K_{3}=17$ (Large)
Core Size	Dynamically adjusted based on scale (e.g., $K_{1}=5$, $K_{2}=9$, $K_{3}=17$)
Learning Rates	Position: 0.00016 → 0.0000016 Feature: 0.0025 Opacity: 0.05 Scaling: 0.005 Rotation: 0.001
Termination Criteria	60,000 iterations, opacity reset every 3000 iterations, densification every 100 iterations

**Table 2:** Training, testing time, and GPU memory usage for different scenarios.

Scenario	Training Time (h)	Testing Time (s)	GPU Memory Usage (%)
Camera Motion Blur	≈1.0	≈6	≈45%
Real Defocus Blur	≈1.5	≈8	≈60%
Mixed Resolution	≈2.0	≈12	≈75%

**Table 3:** Evaluation metrics for camera motion blur dataset.

Method	PSNR	SSIM
NeRF [1]	22.69	0.635
3D-GS [2]	21.66	0.615
Mip-Splatting [53]	21.87	0.627
Deblur-NeRF [14]	25.63	0.768
DP-NeRF [15]	25.91	0.775
PDRF-10 [16]	25.98	0.725
BAGS [22]	26.70	0.824
Ours	27.09	0.885

**Table 4:** Evaluation metrics for real defocus blur dataset.

Method	PSNR	SSIM
NeRF [1]	22.40	0.666
3D-GS [2]	20.57	0.606
Mip-Splatting [53]	21.08	0.623
Deblur-NeRF [14]	23.46	0.720
Sharp-NeRF [18]	23.55	0.724
DP-NeRF [15]	23.67	0.730
PDRF-10 [16]	23.85	0.738
BAGS [22]	23.95	0.754
Ours	23.97	0.883

**Table 5:** Evaluation metrics for mixed resolution dataset.

Method	PSNR	SSIM
NeRFacto [52]	21.90	0.548
Mip-NeRF 360 [9]	26.18	0.721
Mip-Sp [53]	26.11	0.727
Mip-Sp-HAT [54]	24.99	0.603
BAGS [22]	27.11	0.791
Ours	28.30	0.819

**Table 6:** We By sequentially removing modules—GAKA, SAFM, MCA, RGBD, and HPKO—we analyze their impact on performance. Performance is measured using PSNR and SSIM, showing how the combination of modules influences the model’s effectiveness under different blur types. Green checkmarks indicate that a module is included, while red crosses indicate that the module is removed.

Method					Camera Motion Blur		Real Defocus Blur		Mixed Resolution	
**GAKA**	**SAFM**	**MCA**	**RGBD**	**HPKO**	**PSNR**	**SSIM**	**PSNR**	**SSIM**	**PSNR**	**SSIM**
**✗**	**✗**	**✗**	**✗**	**✗**	24.91	0.794	22.65	0.824	27.21	0.756
**✗**	**✓**	**✓**	**✓**	**✓**	26.44	0.861	23.52	0.852	27.87	0.792
**✓**	**✗**	**✓**	**✓**	**✓**	25.44	0.840	23.47	0.864	27.72	0.786
**✓**	**✓**	**✗**	**✓**	**✓**	25.69	0.851	23.62	0.876	27.91	0.796
**✓**	**✓**	**✓**	**✗**	**✓**	25.12	0.843	23.31	0.858	27.67	0.776
**✓**	**✓**	**✓**	**✓**	**✗**	25.27	0.849	23.40	0.861	27.66	0.782
**✓**	**✓**	**✓**	**✓**	**✓**	27.09	0.885	23.97	0.883	28.30	0.819

**Table 7:** Ablation on individual and combined functional components. “MSD-only” uses HPKO alone for deblurring; “RE-only” applies multi-scale adaptive attention for resolution enhancement; “GS-MSDR (full)” combines both.

Configuration	Camera Motion Blur		Real Defocus Blur		Mixed Resolution	
	PSNR	SSIM	PSNR	SSIM	PSNR	SSIM
Baseline	24.91	0.794	22.65	0.824	27.21	0.756
MSD-only	25.60	0.842	23.05	0.846	27.55	0.775
RE-only	25.27	0.849	23.40	0.861	27.66	0.782
GS-MSDR	27.09	0.885	23.97	0.883	28.30	0.819

**Table 8:** Cross-module interaction analysis: performance degradation when removing one module at a time while keeping HPKO active. Values indicate PSNR/SSIM drops relative to full GS-MSDR.

Removed Module	Camera Motion Blur		Real Defocus Blur		Mixed Resolution	
	PSNR	SSIM	PSNR	SSIM	PSNR	SSIM
GAKA	−0.65	−0.024	−0.45	−0.031	−0.43	−0.027
SAFM	−1.65	−0.045	−0.50	−0.019	−0.58	−0.033
MCA	−1.40	−0.034	−0.35	−0.007	−0.39	−0.023
RGBD	−1.97	−0.042	−0.66	−0.025	−0.63	−0.043

## Data Availability

The public sources of the data mentioned in this study are described in the paper.
